# Coping strategies for household water insecurity in rural Gambia, mediating factors in the relationship between weather, water and health

**DOI:** 10.1186/s12889-024-20588-5

**Published:** 2024-11-13

**Authors:** Indira Bose, Robert Dreibelbis, Rosemary Green, Kris A. Murray, Omar Ceesay, Sari Kovats

**Affiliations:** 1 Department of Public Health, Environments and Society, London School of Hygiene & Tropical Medicine, London, UK; 2 Centre on Climate Change and Planetary Health, London School of Hygiene & Tropical Medicine, London, UK; 3 Department of Disease Control, London School of Hygiene & Tropical Medicine, London, UK; 4 Department of Population Health, London School of Hygiene & Tropical Medicine, London, UK; 5 Medical Research Council Unit The Gambia Unit at London School of Hygiene & Tropical Medicine, Atlantic Boulevard, Fajara, The Gambia

**Keywords:** Coping strategies, Water insecurity, Infectious diseases, Seasonality, Food insecurity, The gambia

## Abstract

**Background:**

Rural communities in low- and middle-income countries, such as The Gambia, often experience water insecurity periodically due to climate drivers such as heavy rainfall and reduced rainfall, as well as non-climate drivers such as infrastructural issues and seasonal workloads. When facing these challenges households use a variety of coping mechanisms that could pose a risk to health. We aimed to understand the drivers of water insecurity (climate and non-climate), the behavioural responses to water insecurity and the risks these responses pose to the health of communities in rural Gambia and map these findings onto a conceptual framework.

**Methods:**

We interviewed 46 participants using multiple qualitative methods. This included in-depth interviews and transect walks. A subset of 27 participants took part in three participatory pile-sorting activities. In these activities participants were asked to rank water-related activities, intrahousehold prioritisation of water, and the coping strategies utilised when facing water insecurity.

**Results:**

Multiple strategies were identified that people used to cope with water shortages, including: reductions in hygiene, changes to food consumption, and storing water for long periods. Many of these could inadvertently introduce risks for health. For example, limiting handwashing increases the risk of water-washed diseases. Deprioritising cooking foods such as millet, which is a nutrient-dense staple food, due to the high water requirements during preparation, could impact nutritional status. Additionally, storing water for long periods could erode water quality.

Social factors appeared to play an important role in the prioritisation of domestic water-use when faced with water shortages. For example, face-washing was often maintained for social reasons. Health and religion were also key influencing factors. People often tried to protect children from the effects of water insecurity, particularly school-aged children, but given the communal nature of many activities this was not always possible. Many people associated water insecurity with poor health.

**Conclusions:**

To reduce the risks to health, interventions need to address the drivers of water insecurity to reduce the need for these risky coping behaviours. In the short term, the promotion of behavioural adaptations that can help buffer health risks, such as water treatment, may be beneficial.

**Supplementary Information:**

The online version contains supplementary material available at 10.1186/s12889-024-20588-5.

## Introduction

Many households in low- and middle- income countries, such as The Gambia, are facing increased water insecurity driven by climatic events, infrastructural challenges, seasonal variation and water-management issues [1–3]. Experiencing water insecurity at the household and individual level, defined as the inability to access adequate, safe and reliable water [4], has been associated with a wide range of mental and physical health outcomes, such as diarrheal diseases and psychosocial stress [5–7]. Given the potential interlinkages between weather, water insecurity and health, we sought to understand this relationship among rural communities in The Gambia. In particular, we aimed to explore the behavioural responses to water insecurity and how these responses might reduce or exacerbate health risks.

Previous studies have documented how individuals utilise a variety of mechanisms to either improve their access to or modify their usage of water in response to water insecurity [1, 8, 9]. These can be considered ‘coping strategies’ (behavioural responses when faced with adverse situations, in this case water insecurity) [1]. Many prior studies have found households use coping strategies related to improving the accessibility of water at the household level, such as seeking alternative sources or storing water [1, 8, 10]. Similarly, the strategies used to improve water quality within the household, such as through water treatment, have also been well documented [1, 8, 10]. The behavioural responses on the water-use side that affect how water is utilised at the household or individual level (such as limiting hygiene practices or changing cooking practices), however, has been less explored [1, 9, 11]. Venkataramanan et al., in their review of coping strategies for water insecurity, found that skipping hygiene practices (including hand-washing and bathing) and changing food consumption was mentioned by 8% of studies, respectively [1]. Although this may imply that these are less commonly adopted strategies, this is more likely indicative of the study designs which focus primarily on characterising access-related strategies and might therefore miss some of the strategies related to water-use. The frequency with which people need to rely on these coping mechanisms also remains unexplored. A recent multi-site study identified 19 different coping strategies used by households when faced with water insecurity, however the multiple behaviours on the water-use side are grouped together in two categories: economise; and reduce or change consumption [12]. These categories could encompass a wide range of behaviours (e.g. limiting handwashing, bathing, and laundry, or changing food consumption) that might be important to parse, as they have different implications for health.

The health effects of coping strategies may vary, and some strategies may help to buffer the impact of water insecurity on health. For example, strategies aimed at improving water quality, such as water treatment, could reduce the consumption of poor quality water [1]. Other coping strategies, however, may introduce further health risks [1, 9, 10], as some of these behavioural responses may limit water for hygiene [1], result in contamination of water consumed due to sharing of water sources [13] or have implications for food consumption [14]. Coping strategies are trade-offs that households or individuals must make when faced with water insecurity, so whilst many of these responses may pose health risks, the alternatives may be perceived to be of higher risk. For example, whilst limiting washing hands might have known health risks, people might prioritise water for drinking or cooking as they may consider limiting these behaviours to have more serious health implications. Thus, understanding the decision-making and prioritisation around these trade-offs can help us to design more holistic strategies to protect health in the future.

Coping strategies may vary by context and the nature of the water insecurity faced in a particular area, and a better understanding of these strategies locally is essential. Venkataramanan et al. found that studies looking at coping strategies disproportionately represented Kenya, Bangladesh and India [1]. Whereas some regions such as West Africa remain largely unstudied [1], with the exception of Ghana [15] and Nigeria [16]. There are many countries where people experience periods of water insecurity, but this experience and the strategies utilised in these contexts may vary substantially. Research on coping strategies for food insecurity have highlighted that these can vary according to both exogenous factors (such as local climate, infrastructure and culture), as well as endogenous factors (such as socio-economic status or demographic characteristics of the household or individual and the drivers and severity of the food insecurity experienced) [17, 18]. This demonstrates the importance of capturing these strategies in different populations.

This study focuses on two regions (Kiang West and Basse) in The Gambia, a low-income country in West Africa. We have previously found in this setting that many rural households still regularly face water insecurity, which can be exacerbated by climate drivers (including heavy rainfall and reduced rainfall) and non-climate-related drivers (e.g., mechanical breakdowns and seasonal workloads) [2]. When faced with water insecurity people reported using coping mechanisms to limit water use when faced with these issues [2]. The objectives of this study were thus to further explore the behavioural responses of households when faced with water insecurity and identify if these strategies posed any potential benefits or risks to health. Differences in these behaviours were explored by gender, study site and ethnicity of participant. From these findings a conceptual framework was developed mapping the drivers of water insecurity in the Gambian context, the behavioural responses to these factors, and their linkages to reducing or compounding other health risks.

## Methods

### Study setting

This study was set in rural populations in two regions of The Gambia, Kiang West (KW) and Basse (BA), see map in Supplementary Files Figure S1. The Gambia has two distinct seasons, one short wet season and one long dry season. Basse typically experiences drier and hotter conditions than Kiang West [19]. The Gambia is also experiencing increased variability in its rainfall patterns [20, 21], with these trends expected to continue in the future [22]. Given the importance of rainwater as a supplemental water source in this area [2], this may affect future water access. A higher frequency of heavy rainfall events is also anticipated in the region [23], which may pose a threat to water quality [24]. The Gambia is also classified as being in “food-deficit” [25] and the majority of agriculture is rainfed [26], so changes in rainfall may also have major implications for food insecurity.

In rural areas of The Gambia, access to a basic level of water services (an improved water source where total collection time is no more than 30 min [27]) is fairly high (64%) but access to safely managed water sources remains low (12%) and lagging far behind urban areas (68%) [28]. Based on a survey conducted in 2018, Mansakonko (which is the broader administrative region containing Kiang West) has higher access to WASH facilities than Basse, such as increased access to handwashing facilities at household level (45% vs 24%), and lower indication of faecal contamination of water at the source (36% vs 65%) [29]. In the study communities, we have previously found that most participants had access to a basic level of water service and some had recently (in the last couple of years), gained access to new water sources, such as solar-powered pumps [2]. Many of these participants, however, still regularly faced water insecurity, often seasonally [2]. Participants reported seasonal changes in relation to water access due to both climate and non-climate-related factors [2]. These included seasonal workloads that were higher in the rainy season, cloudy conditions in the rainy season limiting functionality of solar-powered boreholes, and water sources running dry during the dry season [2].

The country has a significant health burden in terms of undernutrition and infectious disease, with malnutrition and enteric infections being ranked as leading causes of disability-adjusted life years (DALYs) lost for children under five [30]. In Basse the prevalence of undernutrition amongst children under 5 is higher than the national average with 21% stunted (national average is 17.5%) but diarrheal prevalence is lower (15.2%, national average is 19%) [31]. In the Kiang West region stunting prevalence is slightly below the national average (16%), but diarrheal prevalence is higher (21.8%) (as indicated by data from Mansakonko) [31]. There is also a clear seasonality in many diseases in The Gambia, often with peaks in incidence during the rainy season [32–38].

### Sample and selection of participants

In addition to one ‘pilot’ village in Kiang West, purposive sampling was used to identify 4 villages in Kiang West (KW) and 4 villages in Basse (BA) for this study. Villages with differing access to water were selected; for example, some primarily used wells and handpumps, whereas others had access to solar-powered boreholes. These villages were selected based on discussions with local experts. Within each selected village, purposive sampling was used to identify women and men in the village that were parents of children under five, as children of this age are highly vulnerable to water-related diseases. In each village three women with children under five, and one man with a child under five were interviewed. We enrolled more women in the study, as in The Gambia women are normally responsible for domestic water collection [29] and tasks involving water within the household, as well as having primary responsibility for childcare. In addition to this, an Alkalo (village chief)/elder was interviewed to provide a wider perspective on water and health in each village. A subset of the total sample (*n* = 27) was asked to participate in pile-sorting activities conducted during the second round of interviews: two of the women interviewed in each village during the first round and one man. Eligible participants were identified through consultations with village officials, and participants were recruited based on their interest and availability.

### Data collection

During the study we used in-depth interviews (IDIs) and participatory qualitative methods (transect walks and pile-sorting). Field notes were also taken throughout of any observations to add contextual understanding.

**Fig. 1:**
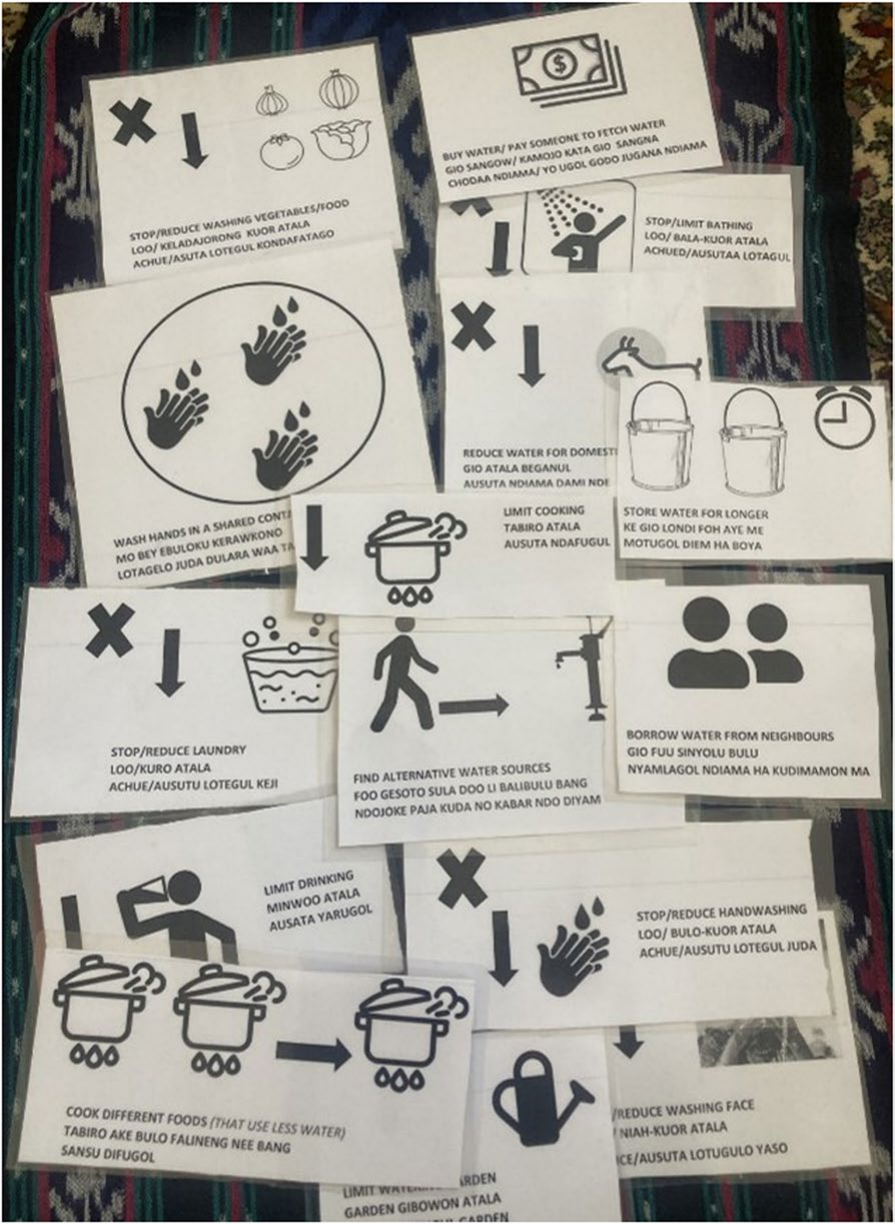
Photo of a selection of cards used during the pile-sorting exercises

Data were collected in two rounds from April to May 2022 at the end of the dry season. In the first-round, semi-structured in-depth interviews and transect walks were conducted only with women using a topic guide. The interviews were conducted by IB (author) and two male field workers from villages in Kiang West, who were fluent in the local languages (Mandinka and Fula). A total of 28 women were interviewed in the first round: 12 in Basse and 16 in Kiang West. For the second round of data collection, emergent findings from the first interviews informed the development of three pile-sorting activities. These activities provided an opportunity to understand how participants perceive certain behaviours, to explore how participants rank or group them in relation to each other, and to prompt further discussion about the factors that influence these rankings/groupings [39, 40]. Picture cards were made to assist in these activities as many of the participants were not literate. Underneath the card was text written in English and phonetic Mandinka and Fula (see Fig. 1 for a photo example of the cards used). The activity and what was written on the card were explained to each participant in their preferred language.

During the first pile-sorting activity, we showed participants cards with different daily water-use behaviours and asked them to rank them based on importance. We also asked participants to clarify if any key water uses were missing. In the second activity we developed cards with different coping strategies employed when there were some water shortages, based on strategies mentioned in the first set of interviews and additional coping strategies reported in the literature. We asked participants to sort these into 3 piles: 1) Frequently adopt these strategies; 2) Sometimes adopt these strategies; and 3) Never adopt these strategies. They were also asked if any strategies were missing and when was the last time, they had to use any of these strategies. In the third pile-sorting activity, we asked participants how they prioritised water amongst household members. During each activity we asked participants to talk us through their ranking and we had a brief discussion after each activity to better understand these behaviours. All topic guides and interview cards can be accessed from the online repository Bose et al, 2024 [41]: https://datacompass.lshtm.ac.uk/id/eprint/3808/.

All interviews (from rounds 1 and 2) were recorded then orally translated into English by the field workers. These were transcribed by IB. The ranking exercises were recorded into an excel database. A selection of these transcripts was then checked by OC for quality control purposes.

### Data analysis

Data from the pile-sorting activities were analysed using Microsoft Excel. Differences between means and medians by geographic area, ethnicity and gender were analysed, and tabulated. During the pilot round, some activities were modified, so data were excluded for one participant on the frequency of the use of different coping mechanisms, as a slightly different approach was used when conducting this activity with the participant.

The discussion around the pile-sorting was uploaded into NVivo [42], along with the transcription of the in-depth interviews, and coded, based on coding developed in the initial reading of the transcripts (see the online repository Bose et al, 2024 [41] for access to the codebook). The information from the pile-sorting activities (PS) was triangulated with information from the in-depth interviews (IDI), field notes and photos from the transect walks (TW) that were coded using a thematic approach [43].

For the first pile-sorting activity related to water prioritisation, four key themes emerged in the initial reading of the transcripts that appeared to influence the order of the ranking: social; health; practicality; spirituality/religion. The explanations for the ranking order were then coded into these themes to understand how these factors may have motivated water prioritisation. For the second activity related to the frequency of using different coping mechanisms, the data were aggregated by the activities described. Data from the interviews with the wider study population were also used to better understand the discussion around the pile-sorting activities and how well this corresponded with the experiences of the wider community. Similarly for the justifications related to intrahousehold prioritisation (the 3rd pile sorting activity) the data from the wider interviews were used to triangulate these descriptions. Finally wider discussions on health were coded to understand what health issues people perceived to be the most common in these communities, the drivers of poor health in this area and how people perceived that this was linked to water-related issues and broader seasonal changes.

Based on these findings a conceptual framework was developed mapping the drivers of water insecurity and the linkages to potential health risks in the Gambian context, to better understand the role that these coping mechanisms may play in mediating the relationship between weather, water and health. In this framework the drivers of water insecurity were grouped into climate impact drivers (based on the definition provided by the Intergovernmental Panel on Climate Change (IPCC) [44]), and non-climate drivers. The physical impacts of these drivers on water insecurity, household behavioural responses to these factors and their linkages to improving or compounding other health risks were mapped in the framework. Food insecurity impacts were also identified by participants, and since these have close linkages with water insecurity, they were also included in the framework. The drivers of water insecurity in this context were identified based on the data collected during the wider interviews, which are presented in the Study setting and explored in more depth in Bose et al., 2024 [2]. The health risks mapped in the framework were identified through examining the epidemiological literature pertaining to each of the behaviours and the associated health implications. For example, there have been numerous studies showing the link between poor hygiene and water-washed diseases [45, 46].

### Ethical approval

Informed consent was given by all participants. Prior to every interview a consent form was read out loud in the preferred language to the participant and the participant provided written consent. Participants who were not literate provided thumbprints and an impartial witness was present throughout the consenting process who also provided their signature or thumbprint. All identifiers were removed during the transcription process and unique identifiers were used to store all files.

This study was reviewed and approved by the Scientific Coordinating Committee of the MRC Unit The Gambia at the London School of Hygiene and Tropical Medicine (LSHTM) and ethical approval was provided by the Gambian Ethics Committee and the LSHTM Observational/Interventions Ethics Committee (Ref: 26,658).

## Results

### Participant characteristics

A total of 46 participants were included in the study, 27 of whom engaged in the pile-sorting activities. Table 1 displays the key socio-demographic characteristics of the respondents engaged in the pile-sorting activities. In Kiang West, the majority of respondents who participated in the pile-sorting were of Mandinka ethnicity (*n* = 10, 67%), and in Basse the majority were Fula (*n* = 9, 75%). These were all predominantly Muslim communities. Detailed demographics on the full study population are outlined in Bose et al., 2024 [2]*.*

**Table 1:** Sociodemographic characteristics of participants in the pile-sorting activities (*n* = 27)

	**All Sites**		**Kiang West**		**Basse**	
	Women	Men	Women	Men	Women	Men
**Ethnicity**						
* Mandinka*	9	4	7	3	2	1
* Fula*	6	5	0	2	6	3
* Other*	3	0	3	0	0	0
**Average Age**	33	44	33	44	32	45
**Average Number of Children**	5	11	6	10	4	13
**Level of Education**						
* No School*	10	6	4	3	6	3
* Some Primary School*	4	0	2	0	2	0
* Completed Primary School or any levels of secondary schooling*	4	2	4	2	0	0
* Arabic School*	0	1	0	0	0	1
**Household Main Income**						
* Farming*	18	8	10	5	8	3
* Business*	0	1	0	0	0	1
**Water Sources**^**a**^						
* Shared water tap in compound*	1	-	1	-	0	-
* Shared water tap in community*	6	-	3	-	3	-
* Handpump*	6	-	3	-	3	-
* Uncovered Well*	5	-	3	-	2	-

^a^Only reported by Women

Most participants described recently experiencing some form of water insecurity, with many facing seasonal challenges in being able to access sufficient water for their domestic needs, although these experiences of seasonal changes varied among participants depending on their primary water source.

*“During dry season to have drinking water, likewise, taking bath is a challenge. Likewise, even if you want to wash your face…”* [IDI, Woman, BA, primary water source- handpump].

*“We have these water shortages in both seasons, but the most difficult [is]… the wet season. This is …because … at times it is so cloudy and we are using these [boreholes powered by] solar panels, so…we do not have enough water.”* [IDI, Woman, KW, primary water source- tap in compound linked to solar-powered borehole].

Due to these challenges in accessing sufficient water, many described having to prioritise their water needs and utilise coping mechanisms when faced with water insecurity.

### Water use prioritization

When asked which household water-use activities they felt were the most important, most participants ranked drinking, cooking and bathing as the highest (as shown in Table 2). Water for sanitation activities and water for domestic animals, along with cleaning the house, washing utensils/basins and washing vegetables/food, were the lowest ranked activities. There were not substantial differences in these rankings by region or by gender, except for water for domestic animals which tended to be more highly ranked in Basse than in Kiang West (see Table 2).

**Table 2:** Ranking of the Importance of Water- Related Activities (Median), broken down by region and gender

	**Median Ranking ***(1* = *1st priority- 14* = *last priority)*				
Activities	Overall (*n* = 27)	Basse (*n* = 12)	Kiang West (*n* = 15)	Woman (*n* = 18)	Man (*n* = 9)
Drinking	**1**	1	1	1.5	1
Cooking	**3**	3	3	3	2
Bathing	**3**	4.5	2.5	3	4
Ablution	**4**	4	7	4	6
Handwashing	**6**	7.5	6	6.5	6
Washing face	**8**	7	8.5	6	8
Laundry	**8**	8.5	7.5	8	8
Brushing teeth	**8**	11.5	7	8	8
Toilet	**9**	8.5	8	7.5	9
Washing vegetables/food	**9**	8.5	8.5	8	9
Cleaning utensils/basins	**9**	9.5	8	10	10.5
Cleaning house	**10**	10.5	9.5	10	10.5
Water for domestic animals	**11**	7.5	12	11	7

The factors that influenced the ranking of these behaviours were: health; social; religious; and practical factors. Good health was often mentioned when explaining the rankings, specifically the importance of hygiene to avoid sickness and infection. This explanation was not made in reference to any particular household members.

*“You will need water to take a bath to wash your body to be clean. It will help you to be hygienic and healthy.”* [PS, Woman, BA].

Social factors also seemed to influence the ranking selections, with many discussing the importance of making sure that their body (including face and mouth) and clothes were clean so that they could go out and socialise with people, and so that others would not think they smelt or were dirty. Some even mentioned the importance of making sure the cooking environment (including cooking utensils) was clean, and the water stored was properly closed, so that their neighbours would not think that they are dirty and would still want to eat and drink at their home.

*“If you are not cleaning your house and mopping it, people can come and start saying that this [person] is very dirty…. Bathing, it is very important,…you have to make sure you are clean. If your body is dirty, if you go out, people will not allow you to sit near them.”* [PS, Woman, KW].

These were predominantly Muslim communities, so when describing water use, many ranked ablution (spiritual cleaning before prayer in Islam) highly. Some also mentioned religious/spiritual factors influencing their ranking, as they mentioned the importance of cleanliness (in terms of body, face and sometimes clothes) in connection with the teachings of Islam.

“*You know as a Muslim, if your body is not clean, your prayer is not going to be proper.”* [PS, Man, BA].

For some participants, practicality seemed to be the factor most influencing their ranking, and as these activities are all routine activities, they were not able to give another explanation for the order of prioritisation. Whilst domestic animals were the lowest ranked overall, some spoke about the importance of providing them with water and watering their gardens as a source of income and food for the family.

“*When you have a garden you can get something out of it that you can bring to the household for food or you can sell it to someone to have money.”* [PS, Woman, KW].

During this pile-sorting activity, three activities were not included in the ranking for some participants, as they were not relevant behaviours for them: watering gardens (*n* = 2, 7%) as they did not have a garden; cleaning the house using water as they swept the floors instead (*n* = 2, 7%); cleaning utensils or basins using water (*n* = 1, 4%). Three participants each identified an additional activity that they felt was not represented within the cards: washing water containers; washing millet; and water for farming (irrigation).

### Coping strategies for water shortages

**Fig. 2:**
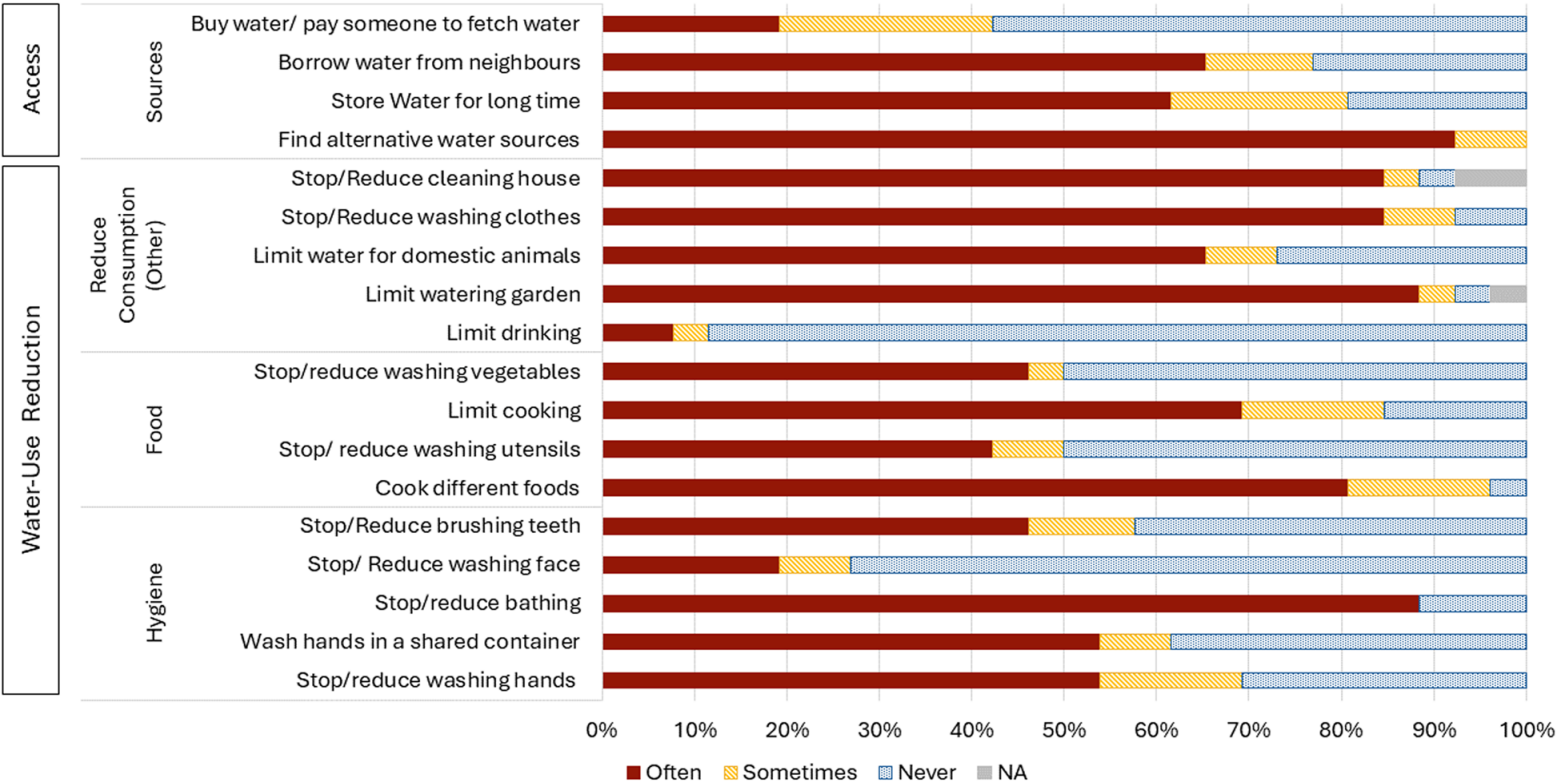
Frequency of coping mechanisms reported in response to water shortages (*n* = 26)

Many households mentioned experiencing water shortages (having insufficient water available in the household for their essential needs) and using a variety of different coping mechanisms to reduce their household water consumption (water-use-reduction strategies) or access the additional water they needed (access-related strategies). Figure 2 presents the results of the pile-sorting exercise where participants ranked how often they used any of these coping mechanisms when they experienced water shortages. There were no major differences observed in the use of these coping mechanisms between Basse and Kiang West, or by gender. There were differences found between Fula and Mandinka communities for some mechanisms, with Fula communities more rarely reducing handwashing and limiting water for domestic animals.

It might be assumed that the order in which participants prioritised water use (displayed in Table 1) would also be inversely reflected in the behaviours which participants modified when faced with water shortages, for example as cooking and bathing were highly prioritised then perhaps we would expect people to very rarely modify or reduce these activities. However, as shown in Fig. 2, this was not found to be the case for many of these activities.

#### Access-related strategies

Households often relied on multiple water sources to meet their demands, so it was quite common to look for alternative sources (*n* = 26, 100%, Fig. 2). Sometimes this entailed using less preferred sources within the village or venturing to other villages. Seeking alternative sources was also the primary coping mechanism employed if water was perceived to be poor quality. Water treatment was not widely practised, so if alternatives could not be found, participants had to use the source they perceived to be dirty. Buying water was also not practised in these areas, but some participants did report paying someone (*n* = 11, 42%, Fig. 2) with a vehicle or a donkey cart to fetch water from far away if they were not able to do this themselves.

*“We always go to other water sources, even yesterday we went up to [a nearby village] to fetch water… we normally pay the donkey carts here [to get the water]”* [PS, Woman, KW].

Borrowing water from neighbours was quite commonplace (*n* = 20, 77%, Fig. 2) with many describing both borrowing and lending water to their neighbours on different occasions. Some even reported that this occurred on the day of the interview, which they would replace when they were able to acquire more water.

*“Yes we do this often. When you are short of water you can borrow a 20L gallon from your neighbour until you have water to repay”* [PS, Woman, KW].

A few described challenges to borrow, as their neighbours would also not have enough to share and even described people hiding the water they had in their house. Hiding water was not commonly mentioned, however, and this situation seemed to be driven by social dynamics within communities, such as tensions with neighbours. Storing water for long periods was a quite commonly used strategy (*n* = 21, 81%, Fig. 2). Some reported that they had to store water for multiple weeks until the point that the smell or taste changed, and one reported sometimes discovering a fungus in the water (slimy texture).

*“We use the water little by little. At times we make it last for 6 days. At times even the odour of the water changes. At times even to drink, you don’t feel comfortable with it.”* [IDI, Woman, BA].

#### Water-use-reduction strategies

Reducing bathing was one of the most commonly used coping mechanisms (*n* = 23, 88%, Fig. 2), and bathing was prioritised more for the children who were going to school rather than others in the household. Some reported that they did not bathe for multiple days due to water shortages. Handwashing was also sometimes deprioritised due to water shortages, in favour of other essential water-related behaviours.

*“How can you think of washing your hands? Our problem is water”* [IDI, Woman, BA].

Some stopped handwashing entirely, whereas others reduced the water used, (*n* = 18, 69%, Fig. 2). Washing hands in one container was another mechanism used to help conserve water (*n* = 16, 62%, Fig. 2). Those that used this method mentioned they had been trained that this was not a hygienic practice, but when faced with water shortages people still had to use this method. Others reported using a cloth to wipe their hands without using any water.

*“What we normally do, there is a cloth, which we normally use for cleaning our hands, so we thoroughly rub our hands inside that cloth and then we start eating. After eating also, we use that same cloth to make sure our hands get clean”* [IDI, Woman, KW].

Washing mouth/ brushing teeth was also sometimes limited due to water shortages, with some forgoing this completely, (*n* = 15, 58%, Fig. 2). Others used a teeth-cleaning twig instead, which was routinely practised by some regardless of the water situation. Washing faces was limited by some participants (*n* = 7, 27%, Fig. 2), but this was the least commonly used strategy linked to hygiene, as it was considered important for social reasons and did not consume much water.

A few participants mentioned moderating the amount of water they drank, but this was not a commonly reported coping strategy (*n* = 3, 12%, Fig. 2), with most saying they would prioritise drinking above other activities.

*“The amount you will drink you will be thinking about, not washing your hands and these other things.”* [IDI, Woman, BA].

Whilst cooking was a highly prioritised activity, changing food-related behaviours was quite commonly mentioned as a coping mechanism (Fig. 2). Cooking foods that consumed less water, was one of the main mechanisms peopled used to try to conserve water (*n* = 25, 96%, Fig. 2). This might be cooking dishes such as “*Benachin*” (a dish similar to jollof rice), porridge or “*Mbahal*” (a rice and fish dish) that could be cooked in one pot, rather than “*Domoda*” (a ground nut stew) or “*Chou*” (meat or fish dish). Participants mentioned that preparing millet consumed a lot of water, so they would choose to cook rice instead. In addition, limiting the frequency of cooking and the food consumed over the course of a day to conserve water was another coping mechanism used quite frequently (*n* = 22, 85%, Fig. 2). Sometimes certain household members were prioritised, usually children, when this led to food shortages.

*“If that happens, we go to this bakery and buy bread for the kids… but.. adults will cope until they have water to cook”* [IDI, Woman, BA].

Washing cooking utensils and cutlery was not commonly reduced, though participants often said they might instead reduce the amount of water they use or just not use cutlery, instead eating with their hands out of a shared bowl. Alternatively, they may limit the amount of water used to clean these items (limiting or stopping *n* = 13, 50%, Fig. 2). Limiting water use was also reported for cleaning vegetables (*n* = 13, 50%, Fig. 2). Reducing cleaning the house was one of the most commonly adopted strategies (*n* = 23, 88%, Fig. 2), and often described as the first activity to be stopped in times of shortage. Also, as many households had dirt floors, water was not always used for cleaning, regardless of the shortages. Reducing laundry was frequently mentioned (*n* = 24, 92%, Fig. 2), but generally participants described wearing other clothes rather than re-wearing dirty clothes. Water provided for domestic animals was also often limited (*n* = 18, 73%, Fig. 2) if water needed to be prioritised for the household, and some spoke of the animals struggling and falling sick due to these water shortages.

*“At times when there is limited water, it becomes difficult for them [the domestic animals] to get water. To the extent that they get sick.”* [PS, Man, BA].

Participants, however, also spoke of the importance of providing enough to sustain their lives and therefore the necessity of also providing water for them. Limiting watering of gardens was a very commonly used strategy (*n* = 24, 92%, Fig. 2).

As well as the coping strategies related to water-use depicted in Fig. 2, reducing water for personal sanitation activities was mentioned by one participant, but they said that they never practised this. It is possible that the stigma surrounding this behaviour led to under-reporting. Two additional activities were identified as coping mechanisms, which were linked to reducing water use at the community level rather than at the household level: limiting water for farming (*n* = 2, 8%) and limiting water for construction (*n* = 1, 4%). However, the same water sources in the village used for household water collection are sometimes used for these activities, so there are some intrinsic relationships between these experiences of water shortages as trade-offs might need to happen between these activities when water is short.

#### Intrahousehold prioritisation when facing water shortages

In addition to having to limit water use at the household level, participants highlighted the importance of prioritising their children when faced with water shortages. In general, both children under 5 and school-aged children were the highest ranked amongst the family members, though some thought that school-aged children needed to be prioritised first as they went to school so needed to be clean and well-presented. The importance of water to help the children concentrate and achieve in school was also highlighted, as their kids are the future leaders.

“*Yes, we share water amongst ourselves. Because of water problems, mostly we consider the kids before us. Yes, even the kids don’t have much, we give little*.” [IDI, Woman KW].

Some participants lived with their in-laws and these elders were also often prioritised due to cultural norms. There was not a strong difference between the prioritisation of men compared to women, but slightly more women ranked themselves as last priority. As many of the behaviours that were limited are communal, such as food-related behaviours and laundry, these intra-household prioritisations seem to be more related to bathing activities.

#### Frequency of experiencing water shortages that led to use of coping mechanisms

Many respondents reported that they faced water shortages that led them to use these strategies described recently (within the last year). This was commonly reported by women (*n* = 15, 83% of women). Some of these activities, in particular, such as seeking alternative sources, limiting washing clothes, borrowing water and changing cooking practices were described as behaviours they had to use on a regular basis. Others, however, said they had not had to use coping strategies for multiple years. This was more commonly reported by men, who largely were not responsible for many of the water-related activities in the household.

### Conceptual framework mapping the drivers of water insecurity, the behavioural responses and the risks to health

**Fig. 3:**
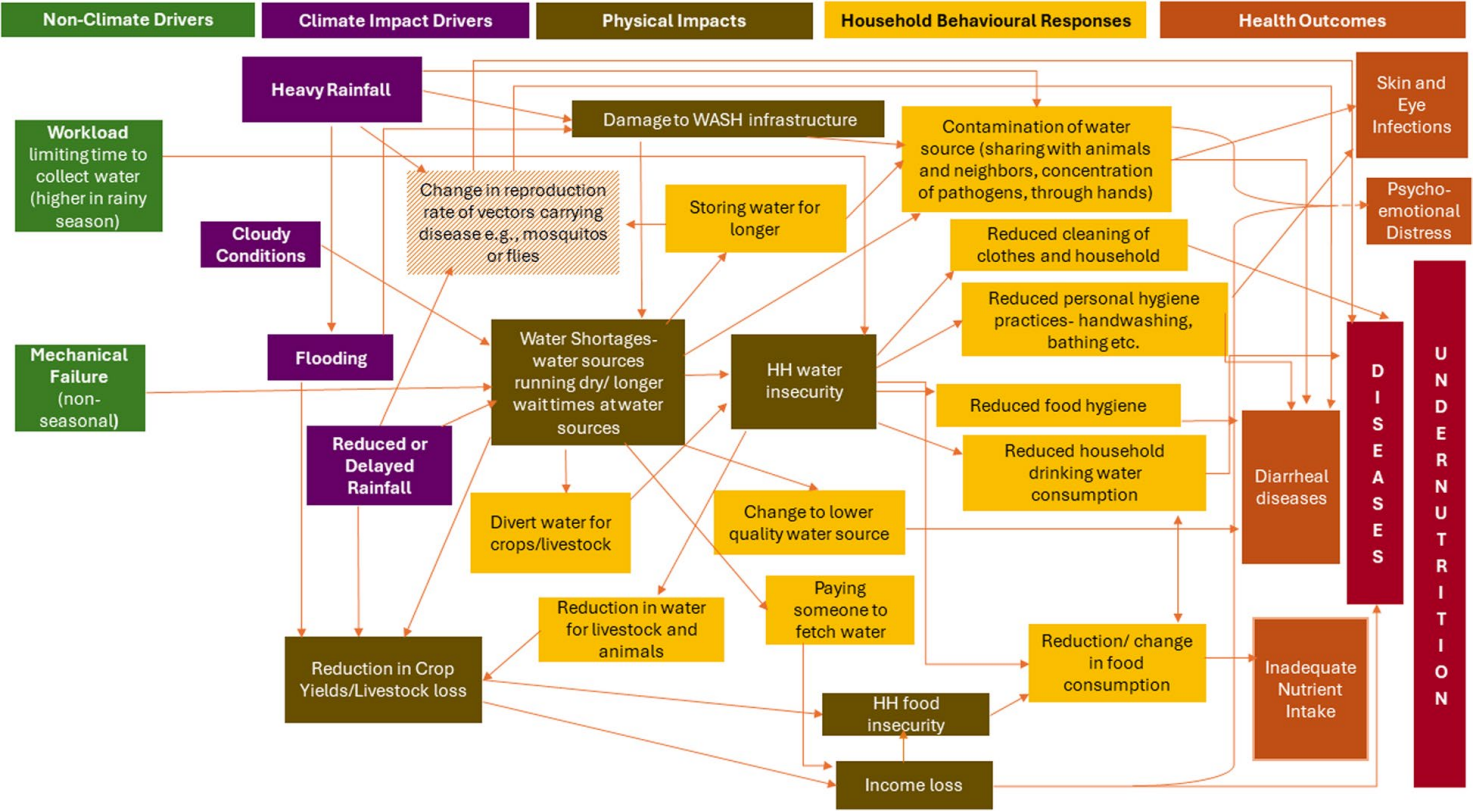
Drivers of water insecurity, the behavioural responses and their risks to health

A conceptual framework was developed (Fig. 3) that maps the driving factors of water insecurity reported by participants in the two areas of The Gambia addressed in this study. Both climate and non-climate-related drivers are shown. Behavioural responses, including but not limited to the coping strategies for water insecurity identified, as well as the risks these responses introduce for health are also displayed. Given the complexity of the full framework in Fig. 3, for visual clarity Figure S2 in the supplementary files provides a snapshot of the framework looking solely at the drivers of water insecurity and Figure S3 provides a snapshot of the framework looking solely at the behavioural responses to water insecurity and the risks these responses pose to health.

This framework depicts multiple factors that can result in people experiencing water insecurity periodically. Many of these factors, such as heavy rainfall, may vary temporally, demonstrating the dynamic nature of water insecurity. Additionally, this framework illustrates the numerous potential health risks associated with the behavioural responses that people described adopting when experiencing water insecurity. From this framework we can also see how closely intertwined people’s experiences are of water and food insecurity, due to the shared drivers of these insecurities and the implications of these behavioural responses for food production, preparation and consumption.

As shown in Fig. 3, many of the coping strategies identified carry health risks and could act as mediating factors in the relationship between water insecurity and poor health. When asked about the diseases that were most prevalent in their communities, many of the diseases identified could have some connection with water, such as malaria (perceived as the most common disease in both communities) and diarrhoea. A strong seasonality was perceived in health, with most participants associating the rainy season with a higher burden of disease.

*“The rainy season has more sickness than the dry season. The dry season is not as bad. In the rainy season if you go to the hospital, you will find it full of kids, women and all are complaining of malaria and diarrhoea. But in the dry season, you will hardly hear that someone is sick.”* [IDI, Woman, BA].

Participants frequently associated issues related to water with common diseases in the area. Poor quality water, water perceived as coming from a source that was not clean, or drinking water that had a strange taste, smell or colour were factors associated by participants with diarrheal diseases, stomach aches and vomiting. Some described how these issues had decreased since interventions to improve access to higher quality water sources were installed, such as boreholes.

*“Since we started drinking this borehole water, diseases like diarrhoea and stomach-issues have reduced in the village”* [TW, Woman, BA].

Participants also attributed some illnesses (diarrheal diseases and stomach issues) to water shortages due to limitations in the ability to practice some hygiene-related behaviours. The stigmatising nature of water shortages was also described by some participants.

*“Water shortage you have lots of problem out of it. Even by looking at you, people will know that you are out of water. As the way your body hygiene should be and the way you are will be different. That dirtiness can cause lots of sickness.”* [IDI, Woman, KW].

This stigma and overall worry related to water insecurity and having to use coping mechanisms may put individuals under great stress and could lead to psycho-emotional distress. The evidence from the literature on the health risks associated with the coping mechanisms identified in Fig. 3 are discussed further in the Discussion section.

## Discussion

In this study we found that many participants had recently experienced water shortages. Due to these shortages, participants often utilised coping strategies to both increase water accessibility at the household level and reduce water usage, with many having to make difficult decisions on how to prioritise water use among their essential routine domestic behaviours. Several of the coping strategies identified pose potential risks to health.

Four key groups of factors were found to influence how participants ranked their water-related activities in terms of importance: health; social; religious; and practical factors. The rankings, however, did not always reflect whether these activities were frequently reduced, limited or adjusted when faced with water shortages. The main exception was drinking water, which was ranked the most important use of water and was a rarely reduced behaviour when water was limited. Cooking and bathing, on the other hand, were amongst the two highest ranked priorities; however, both of these activities were amongst the most common behaviours to be modified during periods of water insecurity. Face-washing was not very highly ranked in the prioritisation, but this was rarely limited, partially as this activity requires only small amounts of water but many were also reluctant to limit this activity owing to social factors. This suggests the importance of social factors, which may take precedence over health factors, as was found in a study in a low-income urban community in Bangladesh [47]. Interestingly the prioritisation of water-use activities in these communities mostly followed a similar pattern to the findings from a study conducted among pregnant and postpartum women in Kenya [11]. Although there were some exceptions, for example washing dishes was ranked more highly in Kenya [11]. This similarity suggests that there may be some generalisability to how people prioritise water across settings. However, our study highlights the disparity that can exist between theoretical prioritisation of water use and the actual frequency of adopting related coping strategies, which may still differ depending on context.

Our findings demonstrate the numerous coping strategies that households adopt when faced with water shortages, many of which pose a risk to health (as shown in Fig. 3). These strategies may therefore act as mediators in the relationship between water insecurity and health. Some of the coping strategies identified that posed key health risks were strategies that aimed to reduce water use at the household or individual level. These strategies and the associated health risks have not been explored in depth in previous studies, only 8% of studies found in a review by Venkataramanan et al. mentioned skipping hygiene practices (including hand-washing and bathing) [1]. Whilst this suggests these coping strategies are adopted fairly infrequently, this may vary greatly by setting and these behaviours may not be adequately captured in some studies. In the communities studied we found that 69% of participants mentioned sometimes, or often, reducing or stopping handwashing. Additionally, 88% of participants mentioned often reducing bathing, which demonstrates the importance of ensuring studies try to capture these behaviours, particularly given the health implications. The reduction in hand-hygiene behaviours described by many participants, including limiting handwashing or washing hands in one container, is a major risk factor for diarrheal diseases, and other water-washed diseases, such as some skin and eye infections [45, 46]. Reducing bathing could also reduce overall hygiene and lead to disease [48]. Oral hygiene practices using water were reduced fairly often, but given the widespread use of teeth-cleaning twigs/sticks, which have been shown to be quite effective [49], this may not have major effects on dental health. Other potentially risky coping strategies identified related to water-use include reducing cleaning the house and washing clothes, which over long periods could reduce the hygiene of the environment and pose a risk to health. However, most participants did not describe having to re-wear very dirty clothes and had alternative methods of cleaning the house, so this is perhaps not a major risk in this setting.

There was a strong inter-relationship found between food and water security, with many participants describing that they changed the types of foods that were cooked when water was limited, and sometimes reduced cooking. Further study is required to assess whether these changes are to foods of lower nutritional value. Although many participants emphasised that millet was a very water intensive food, so they switched to rice during water scarce periods. Millet is more nutritionally dense than rice in a number of different micronutrients, such as iron [50, 51], so it is possible that this switch could have nutritional implications where it is not supplemented by other foods. Limiting food hygiene practices was sometimes used as a coping mechanism, and it was common practice to consume food in a shared bowl using hands, which could increase exposure to pathogens if hand hygiene was also limited at that time. This can also contribute to poor health as hygiene is closely associated with diarrheal diseases and may result in low nutrient absorption or inflammation [52], which can lead to undernutrition. Reducing watering gardens, and the water provided for domestic animals, are also commonly used strategies that could pose health risks. Both gardens and domestic animals are important sources of income for some of these households, so this could limit their household resources and have future implications for their health. Gardens are also sometimes used to supplement household food so this might limit future vegetable consumption, and nutritional status in the longer-term. The linkage between food and water has been found in various other settings [1, 11, 53, 54]. These findings reinforce the importance of considering water insecurity in tandem with food insecurity when trying to improve nutrition.

Commonly utilised adopted coping strategies aimed at improving the accessibility of water at the household level, such as searching for alternative water sources, also pose potential health risks. Whilst this may help to buffer the effects of shortages, it may at times result in water being sourced from lower quality sources and limit time for other activities. Further study is required to measure this, as it is possible that this may also result in higher quality sources being used, as some participants then sourced water from outside of their communities which sometimes came from boreholes that might be safer than their primary water sources. Prior studies examining seasonal changes in the water sources used in different countries, found the impacts these changes have on the quality of the source can vary highly by setting [55, 56]. Some participants also reported having to pay people to help them fetch water, which may reduce the income available for other essential items, such as food. Another key mechanism used was storing water for long periods and many participants noticed a reduction in water quality over this time, which could pose a risk to health particularly as appropriate water treatment is not widely practiced. Studies in Mozambique [8] and Ecuador [57], have shown that longer storage times can facilitate contamination. In The Gambia, a prior survey has found a high increase in contamination that occurs between the source and water stored at the household level [29], and a study in Basse has also found that higher Cryptosporidium-positive diarrhoea in children under five was associated with consuming water stored in the house for two weeks and water filtered through a cloth [58]. These findings suggest that increasing storage time is a key risk to health in this setting. Furthermore, if water storage containers are not properly covered, these containers could also become vector-breeding sites, facilitating the spread of diseases carried by mosquitoes [9], e.g. malaria.

Borrowing water from neighbours, another key mechanism used to improve water accessibility in households, has been shown to have both potential negative and positive effects on health [13]. Whilst borrowing water can buffer access and prevent households from having to limit other behaviours, sharing water may increase the exposure of the water to contamination [13]. Beyond physical health, however, sharing water has been found to have great social significance [59]. In this setting we also found that the ability to benefit from this practice seemed to be governed by social dynamics. Whilst in some cases this practice has been shown to improve solidarity in the community, it can also place great stress on both the givers and the receivers of the water and contribute to poor mental health [60]. More broadly, experiencing water insecurity and the need to use any of these coping strategies, could cause anxiety and shame, which may have a major toll on mental health. This strong link between water insecurity and psycho-emotional distress has been found in numerous other settings [6, 61, 62].

Participants also tried to prioritise water within the household at times, giving preference to children, particularly those going to school. However, as many of the riskiest behaviours identified, such as changing the foods consumed, limiting hygiene (e.g. washing hands in one container) or storing water for long periods, are collective activities it is unlikely that this prioritisation helps buffer this group from some of the risks to health. Contrary to findings in other settings [11, 63] there was not a strong gender difference identified in how water was prioritised at the intrahousehold level, although women seemed to slightly deprioritise themselves.

The conceptual framework in Fig. 3 displays the multifaceted relationship between the drivers of water insecurity (climate and non-climate related), the behavioural responses and health in these communities in The Gambia. In this setting, and more widely, climatic drivers such as seasonal rainfall and extreme events such as flooding can have major impacts on water insecurity [2, 3], which may exacerbate these challenges and thus increase the risks to health. Notably, there are broader health risks that are not portrayed in the diagram, which were raised by the participants. This includes injuries brought about by heavy rainfall and flooding or carrying water back from long distances. Prior studies have also found that obtaining water from more distant water sources can have implications for physical health, as carrying the water back from long distances can result in injuries [10, 64, 65]. A few participants mentioned that water issues resulted in their children being late to or sometimes entirely missing school, which may also have broader effects on health and well-being [66].

Although complex, the framework presented in Fig. 3 allows us to consider more holistically the trade-offs that occur at the household or individual level when faced with water insecurity and the linkages with health, as well as to consider the different drivers of water insecurity. Understanding the hierarchy of needs and the trade-offs that occur in different settings has long been identified as a critical step in the design of food security interventions to improve resilience and mitigate adverse effects [17], and this study indicates that this is also true of water insecurity. Many of the coping strategies identified may not be perceived to be health risks in this setting or might be considered to be of lower risk than alternative strategies. In order to effectively mitigate the risks to health it is important to take this all into consideration in the design of policies and programmes. Greater efforts are required to address the drivers of water insecurity, such as through investment in climate-resilient water infrastructure and maintenance, to reduce the need for these coping mechanisms and help mitigate these health risks. In the short-term promoting coping strategies that might be beneficial for health, such as the promotion of affordable water treatment options may help to address some of the challenges related to water shortages, as well as water quality challenges. If water is adequately treated then some of the health risks related to using alternative sources that may be of lower quality, borrowing water or storing water for long periods could be reduced. Interventions to improve water quality at the household level have been shown to be effective in improving health even when it is not possible to improve water and sanitation infrastructure [67].

### Strengths and limitations

A major strength of this study is the use of multiple methods to explore in depth the relationships between weather, water and health. The pile-sorting exercises enabled the quantification of information that came out of the in-depth interviews to provide a broader perspective on how widespread these practices were in these communities and to probe on these issues more deeply. Our study also presents novel findings on the frequency of different coping strategies, which was largely unexplored in the prior literature. Further it demonstrates that even in settings where people have high access to improved water sources there still are major challenges to water security, and people still need to sometimes adopt coping mechanisms that may pose risks to their health.

There are, however, some limitations. This study is based on 46 participants in The Gambia, 27 of whom were engaged in the ranking exercises. The focus was on households with children under 5 and women were oversampled in this study due to their role within the household related to water. Therefore, these findings cannot necessarily be generalised to represent the experiences of others in different contexts or even in the wider country. Prior studies in other settings, for example, have found that water insecurity can lead to gender-based violence and intimate partner violence, with a large base of literature reporting that travelling long distances for water and inadequate household water supplies are risk factors for experiencing violence [68]. These issues were not raised during the interviews, perhaps indicating that these experiences related to water insecurity differ in this setting or potentially may have not come up given the sensitive nature of the topic and as this was not a key line of inquiry.

The study was qualitative in nature using interviews and pile-sorting techniques, and no measurements were taken to assess water shortages, quality, changes in water use or the health burden. Further study is also required to assess the associations between these coping strategies and health. This study was conducted in the dry season, with the ranking activities taking place shortly after Ramadan and in-depth interviews taking place during Ramadan, which may have influenced how people thought about water. The participants were asked to remember past events, which may have also led to recall bias. The composition of the team, including a foreign woman and male MRC staff, may have influenced the responses. Additionally, the affiliation with the MRC may have also influenced the responses, especially as the MRC has previously provided health interventions in these areas. To reduce this risk as much as possible we tried to explain the study purpose very clearly and that no further interventions were planned. The repeated visits and multiple methods used should have also helped to mitigate some of this risk by building a rapport with participants and enabling the triangulation of the findings.

## Conclusion

Households in rural Gambia use a wide variety of coping mechanisms when faced with water insecurity, such as reducing hygiene or changing food consumption patterns. Social factors, above health, appear to play a primary role in the adoption of some of these behaviours. Many of these behaviours are quite risky for health, particularly for young children and vulnerable groups. Although most people tried to buffer the impacts on children, many of these behaviours are communal and still likely to pose a risk to their health. As some drivers of water insecurity, such as changes in weather patterns, are projected to increase in the future, further efforts must be made to help mitigate future risks to health.

## Supplementary Information

Supplementary Material 1.

## Data Availability

This research was based on qualitative interviews and pile-sorting excercises conducted during these interviews. Excerpts of transcripts to support the analysis are provided throughout the paper. The data from the pile-sorting excercises have been summarised and presented in tables and figures in the paper. The codebook, interview guides and cards used for the pile sorting excercises are available in a repository referenced in the manuscript: https://datacompass.lshtm.ac.uk/id/eprint/3808/. From these documents it is possible to replicate the study. Due to privacy concerns, as this study was conducted in a small population, making the full transcripts available could make participants identifiable and breach their confidentiality. Thus, excerpts of anonymized transcripts and de-identified pile-sorting frequencies may be requested directly from the corresponding author or from researchdatamanagement@lshtm.ac.uk. This will be shared if we can do so whilst still protecting privacy in accordance with the ethical approval obtained from the Gambian Ethics Committee and the LSHTM Observational/Interventions Research Ethics Committee (Ref: 26658).
