# Supplementary material for: Coping strategies for household water insecurity in rural Gambia, mediating factors in the relationship between weather, water and health
Source: BMC Public Health. 2024 Nov 13;24:3150. doi: 10.1186/s12889-024-20588-5 (PMC11562698; doi:10.1186/s12889-024-20588-5)
Supplement: Supplementary file 1 — Supplementary Material 1. [file 12889_2024_20588_MOESM1_ESM.docx]

**Coping Strategies: Supplementary Files**

**Figure S1: Map of Research Sites***


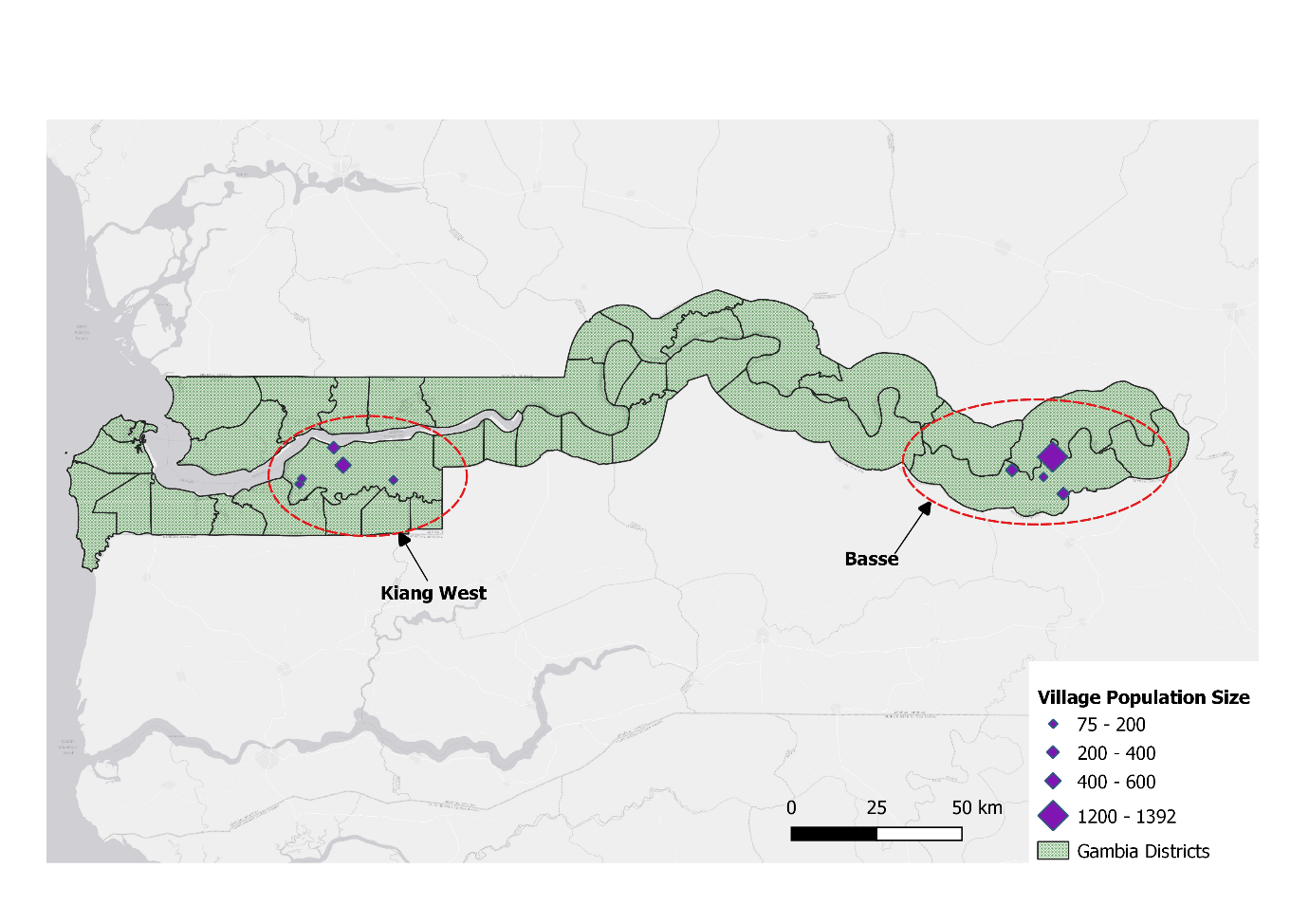


**This map was made in QGIS base layers extracted from the following databases: Gambia National Disaster Management Agency (NDMA) “Gambia—Subnational Administrative Boundaries” https://data.humdata.org/dataset/cod-ab-gmb? Accessed 2024-03-06.; ESRI “World Light Gray Base* [*https://server.arcgisonline.com/arcgis/rest/services/Canvas/World_Light_Gray_Base/MapServer Accessed 2024-03-06*](https://server.arcgisonline.com/arcgis/rest/services/Canvas/World_Light_Gray_Base/MapServer%20Accessed%202024-03-06)*.*

*
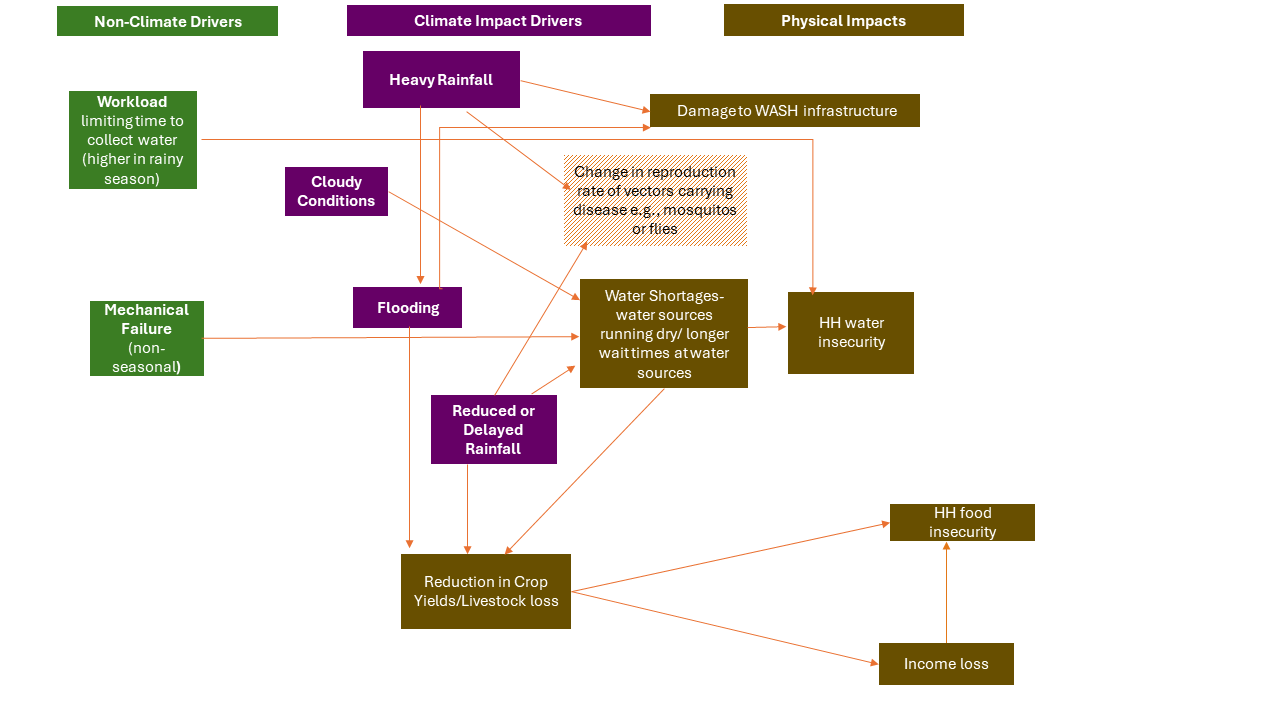
***Figure S2: Conceptual framework- drivers of water and food insecurity only**

*
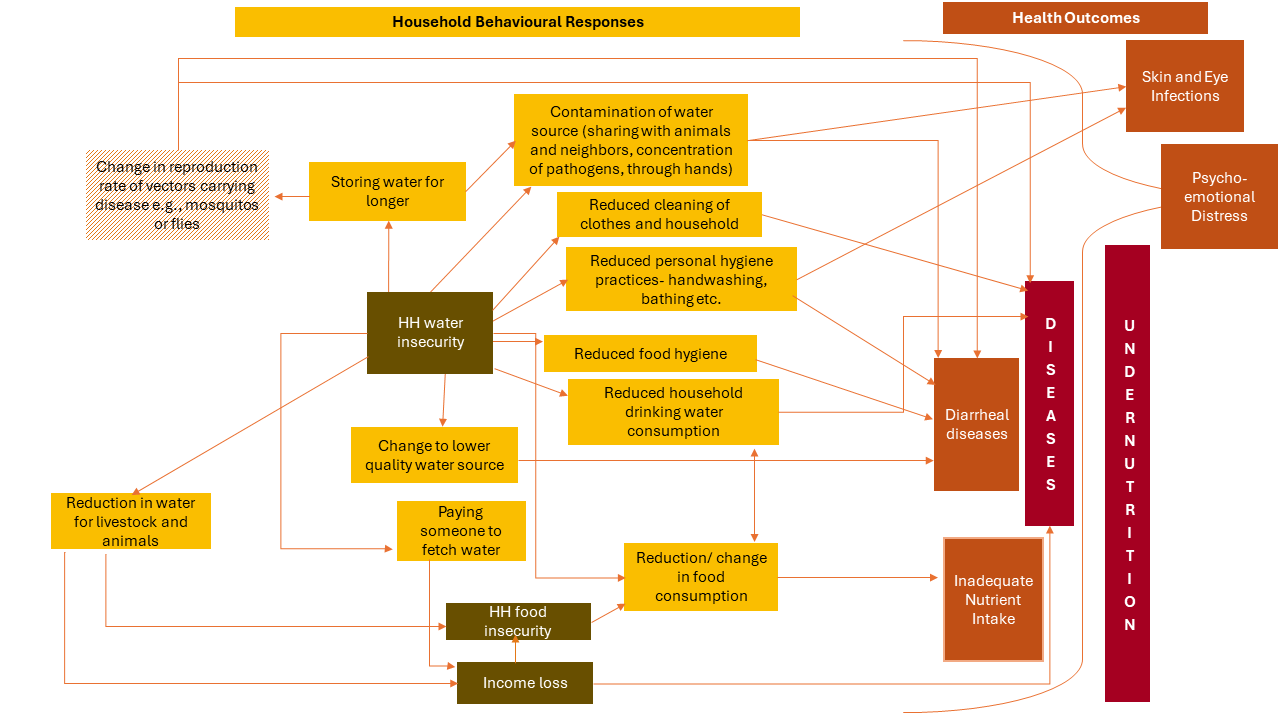
***Figure S3: Conceptual framework drivers- the behavioural responses to water insecurity and their risks to health only**
